# Refining selection signals in dairy sheep using high-density genotyping data

**DOI:** 10.1093/jas/skag055

**Published:** 2026-02-22

**Authors:** Slim Ben Jemaa, Gabriele Senczuk, Corrado Dimauro, Baldassare Portolano, Alberto Cesarani, Salvatore Mastrangelo

**Affiliations:** Dipartimento di Scienze Agrarie, Alimentari e Forestali, University of Palermo, Palermo, 90128, Italy; Institut National de la Recherche Agronomique de Tunisie, Université de Carthage, Ariana, 2049, Tunisia; Dipartimento di Agricoltura, Ambiente e Alimenti, University of Molise, Campobasso, 86100, Italy; Dipartimento di Agraria, University of Sassari, Sassari, 07100, Italy; Dipartimento di Scienze Agrarie, Alimentari e Forestali, University of Palermo, Palermo, 90128, Italy; Dipartimento di Agraria, University of Sassari, Sassari, 07100, Italy; Animal and Dairy Science Department, University of Georgia, Athens, GA, 30602, USA; Dipartimento di Scienze Agrarie, Alimentari e Forestali, University of Palermo, Palermo, 90128, Italy

**Keywords:** selection signatures, high-density array, sheep, genetic diversity, local adaptation

## Abstract

We previously identified broad candidate regions under selection in three ecotypes (plain, hill, and mountain) of Sarda and Valle del Belice sheep across altitudinal gradients using medium-density SNP chips. Here, we employed high-density genotyping data from independent animals to validate and refine these regions, focusing on adaptive signatures in the mountain ecotype. Joint analyses of the three ecotypes confirmed selection signals on chromosomes 19 and X in Sarda and on chromosome 3 in Valle del Belice. In Sarda, five genes were identified, including *KDM6A*, a key regulator of mammary function and stress response. In Valle del Belice, *KCNA5* and *KCNA6* (voltage-gated potassium channels) and *GALNT8* (involved in glycosylation and immune regulation) emerged as candidates linked to cardiac and neuronal electrophysiology and health traits. We found little overlap between the candidate regions identified by the two approaches. Between-ecotype comparisons further confirmed and refined selection signals in the mountain ecotype, particularly on chromosome 3 in both breeds. We identified several missense, synonymous, and intronic variants within genes involved in the regulation of neuroendocrine, nervous, and cardiovascular systems, as well as immune response, respiratory efficiency, and musculoskeletal development, highlighting the multifaceted adaptations of the mountain ecotypes of both breeds to mountainous environments. Overall, our high-density analyses corroborate previous findings from the medium-density chip and, in several cases, refine the candidate regions detected. Although the specific genes under selection differ between the mountain ecotypes of Sarda and Valle del Belice sheep, they converge on similar biological pathways and functions, suggesting parallel adaptive mechanisms to high-altitude conditions.

## Teaser

Comparative genomic analyses of three ecotypes from two dairy sheep breeds living across different altitudinal gradients revealed signatures of selection in genes associated with musculoskeletal development, cardiovascular and immune function, neuroendocrine regulation, and DNA repair. These biological pathways likely contribute to adaptation to high-elevation environments in the mountain ecotypes of both breeds.

## Introduction

Understanding the genetic mechanisms underlying local adaptation is a central objective in animal genomics, with important implications for livestock resilience, productivity, and conservation. Genomic signatures of selection in domesticated animals can reveal how populations have adapted to natural as well as artificial selection targets, particularly in diverse and challenging environments (e.g., Ben-Jemaa et al. 2023; Mastrangelo et al. 2023; Somenzi et al. 2024; Peng et al. 2024).

Local sheep breeds, such as the Sarda and Valle del Belice, represent valuable models for investigating adaptation processes because of their long-standing exposure to distinct ecological conditions and production systems across altitudinal gradients. Previously, we discovered selection signatures in these two dairy breeds with a medium-density SNP array and implicated candidate genomic regions to be likely associated with environmental adaptation and dairy-related traits (Ben Jemaa et al. 2025). However, medium-density genotyping platforms may lack the resolution needed to detect fine-scale selection signals or to accurately localize candidate genes (Zhao et al. 2015; Muñoz et al. 2019).

In this follow-up study, we build upon our previous work by analyzing high-density SNP data (600K array) generated from newly genotyped animals of the Sarda and Valle del Belice sheep breeds. The increased marker density not only enables us to validate earlier findings but also enhances the power to detect novel selection signals and candidate genes.

Using extended haplotype-based methods (iHS and Rsb), we examine both within-breed and between-ecotype variation, considering the influence of environmental gradients (e.g., plains vs. mountains) and the combined effects of natural and artificial selection. Our findings contribute to a deeper understanding of the genetic architecture of adaptation in local livestock populations and highlight the importance of conserving genetic diversity within traditional breeds, particularly as climate change intensifies the need for resilient and locally adapted animals. Moreover, this study serves as a methodological validation by confirming the selection signatures previously identified using lower-density SNP arrays.

## Materials and methods

All experimental procedures and sampling were approved by the Bioethics Committee of the University of Palermo: protocol code UNPA-CLE–203098. Blood samples were collected in compliance with European rules (Council Regulation [EC] No. 1/2005 and Council Regulation [EC] No. 1099/2009) during routine health control by the public veterinary service.

### Animals and genotyping

Blood or nasal swab samples were collected from 135 Sarda (SAR) and 112 Valle del Belice (VDB) ewes representing the three ecotypes: mountain (_M), hill (_C), and plain (_P). Procedures for ecotype classification, animal sampling and DNA extraction are detailed in Ben Jemaa et al. (2025).

Samples were genotyped using the Ovine Infinium HD BeadChip 600 K (Illumina Inc. San Diego, CA, USA) according to the manufacturer’s protocol. Chromosomal coordinates for single nucleotide polymorphisms (SNPs) were updated to the ARS-UI_Ramb_v2.0 (*Ovis aries*) genome sequence assembly. Prior to analyses, we conducted a series of quality control procedures via PLINK ver. 1.09 (Purcell et al. 2007). Samples with genotyping rates <90% were excluded. SNPs were filtered out based on the following criteria: call rate <90%, minor allele frequency (MAF) <5%, or deviation from Hardy–Weinberg equilibrium (*P* < 0.001).

After applying these filters, 478,683 SNPs were retained for 45 SAR_C, 47 SAR_M, and 43 SAR_P individuals, while 492,877 SNPs were retained for 46 VDB_C, 44 VDB_M, and 22 VDB_P individuals. These datasets were subsequently used for selection signature analyses.

### Detecting signatures of selection

Two extended haplotype homozygosity (EHH)-based tests, iHS (Voight et al. 2006) and Rsb (Tang et al. 2007), implemented in the *rehh* package in R (Gautier et al. 2017), were used to detect genomic regions under putative selection in the SAR and VDB breeds. Haplotype reconstruction from genotyped SNPs was performed using fastPHASE version 1.4 (Scheet and Stephens 2006). For each breed, 10 random starts and 40 iterations of the EM algorithm were used, with 50 haplotypes sampled from the posterior distribution. The number of clusters for cross-validation was set to 30. Analyses were conducted at two levels. First, iHS was calculated within each breed by treating the three ecotypes as a single population and then independently for each ecotype (plain, hill, and mountain). The Rsb statistic was used to compare the three ecotypes within each breed, with a specific focus on the comparisons between plain and mountain ecotypes in both breeds because we were primarily interested in finding areas under selective pressure that are associated with environmental adaptation to mountainous environments. Because iHS requires information on ancestral and derived allele states, the ancestral allele was inferred as the most frequent allele in the dataset (e.g., Deng et al. 2025). Selection signature detection was performed using 200-kb sliding windows with a 10-kb overlap. A window was classified as putatively under selection if it contained at least six markers exceeding the significance threshold of −log10 (*P* value) = 3.

### Functional impact of the variants under selective pressure

Ensembl Variant Effect Predictor (VEP) (McLaren et al. 2016) was used to predict the statistically significant variants with the relevant genomic regions putatively under selection. VEP was used to determine the location of the variant (e.g., intronic, intergenic, in a coding sequence, in regulatory regions) and the impact rating (high, moderate, low, or modifier), indicating the severity of the consequences of the mutation.

## Results and discussion

Previously, we performed a genome-wide scan to detect signatures of selection in the three ecotypes of SAR and VDB by analyzing a dataset of 524 individuals genotyped with the OvineSNP50 medium-density BeadChip (Ben Jemaa et al. 2025). We identified several genomic regions, most notably on chromosome 3, under selective pressure in the mountain ecotypes of both breeds. Within these regions, we detected significant variants in genes associated with nervous and neuroendocrine system functions, body size regulation, energy balance, and muscle development and function.

Although medium-density SNP arrays have been widely used to detect selection signatures and genomic regions associated with adaptive traits, their limited marker resolution may lead to incomplete or less accurate identification of candidate genes. In contrast, high-density arrays provide a finer genomic coverage, enabling more precise identification of selection signals and validation of candidate genes and regions. Therefore, in this study, we sought to confirm and refine our previous results by analyzing a different panel of Sarda and Valle del Belice individuals using the Illumina OvineSNP600 BeadChip. High-density genotyping arrays represent powerful tools for genomic studies, providing comprehensive genome-wide coverage and high analytical throughput (Kranis et al. 2013; Carta et al. 2026).

### Selection signature analysis within breeds

The iHS test conducted jointly for the three ecotypes of each breed revealed 15 and 6 outlier windows distributed over four chromosomes in SAR and VDB, respectively (Table S1). In SAR, we detected three strong signals on OAR19 (34.86–40.34 Mb) and on the X chromosome (39.93–45.11 and 51.02–55.51 Mb), with at least 26% of SNPs exceeding the significance threshold. In VDB, we identified several adjacent candidate regions on OAR03 (205–212 Mb), overlapping with those previously detected on this chromosome using the 50K beadchip. Table 1 shows the list of protein-coding genes, including the variants with the highest −log(*P* values). All these regions have been previously reported using the medium-density chip (Ben Jemaa et al. 2025). Regarding the Sarda breed, we identified 18 genes containing the variants with the highest significance levels. Among these, eight genes (*PRICKLE2, SYNPR, CADPS, IQSEC2, SMC1A, USP9X, MAOA*, and *DDX3X*) are implicated in neuronal development, synaptic function, and synapse formation. Five genes (*THOC7, DDX3X, USP9X, KDM6A*, and *GNL3L*) are involved in the regulation of gene expression through transcriptional control and chromatin modification, while four genes (*MAGI1, ADAMTS9, PTPRG*, and *GPR34*) participate in cell–cell interactions, extracellular matrix remodeling, and tissue organization. Notably, five of the 18 genes (*MAGI1, PRICKLE2, SYNPR, PTPRG*, and *KDM6A*) also contained SNPs that were statistically significant with the medium-density SNP chip (Ben Jemaa et al. 2025). The first four of these genes are associated with neuronal function and synaptic organization. The *KDM6A* gene, in addition to its role in epigenetic regulation, controls luminal milk-secreting cells in the mammary gland and may therefore influence milk production traits (Yoo et al. 2016). Moreover, *KDM6A* acts as a key epigenetic regulator modulating gene expression in response to environmental stress (Chen et al. 2021).

**Table 1:** Genes containing the most statistically significant variants (−log(*P* value) >4) within the relevant candidate regions revealed by the iHS test in Sarda (SAR) and Valle del Belice (VDB) breeds.

Breed	Number of SNPs	Location	Genes
**SAR**	158	OAR19: 35.35–39.91 Mb	*MAGI1, ADAMTS9, PRICKLE2, THOC7, C3orf49, SNTN, SYNPR, CADPS, PTPRG*
**SAR**	148	OARX: 40.12–45.02 Mb	*USP9X, DDX3X, GPR34, MAOA, KDM6A, DIPK2B*
**SAR**	4	OARX: 51.02–55.51 Mb	*IQSEC2, SMC1A, GNL3L*
**VDB**	11	OAR03:210.68–212.04 Mb	*KCNA5, KCNA6, GALNT8*

In VDB breed, we identified two genes, *KCNA5* and *KCNA6*, located within the candidate region previously reported by Ben Jemaa et al. (2025), that harbor statistically significant variants in the HD chip analysis. The selection signals identified in these two genes likely reflect adaptive physiological processes underlying the resilience of the breed. Given that *KCNA5* is essential for atrial repolarization (Yang et al. 2009) and *KCNA6* regulates the neuronal resting membrane potential in the central and peripheral nervous systems (Glazebrook et al. 2002), both genes are involved in maintaining proper cardiac and neuronal electrophysiological function. Selection on these loci could therefore be linked to improved stress tolerance under the Mediterranean environmental conditions. The third gene, *GALNT8*, belongs to the GALNT family, which encodes enzymes responsible for O-glycosylation, the addition of sugar moieties to proteins to form O-glycans. These carbohydrate structures are key modulators of host–pathogen interactions and immune responses (Magalhães et al. 2021). *GALNT8* has been previously reported in a genome-wide scan for signatures of selection associated with milk production traits in dairy goats (Peng et al. 2025). Moreover, other members of the GALNT family (*GALNT4, GALNT6*) have been implicated in resistance to gastrointestinal parasite infections in sheep (Benavides 2015; Al Kalaldeh et al. 2019).

### Detecting variants associated with mountain environment adaptation

**Figure 1:**
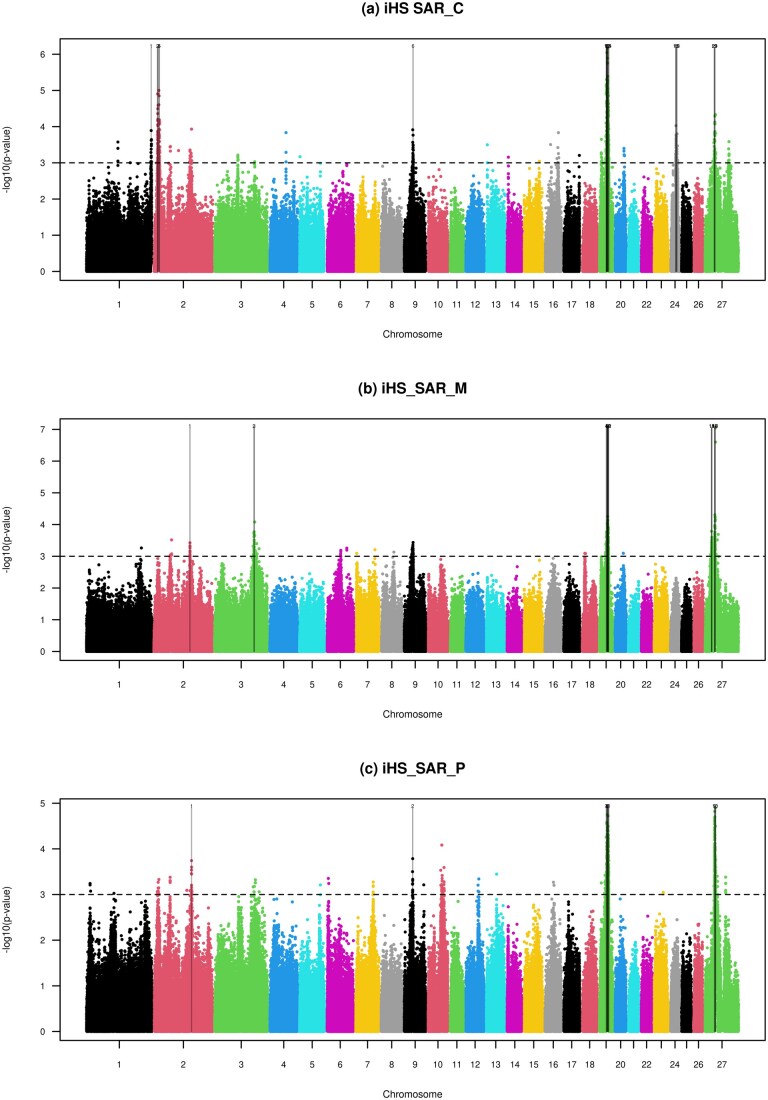
Manhattan plots of the genome-wide iHS test of the three ecotypes of Sarda. The horizontal dashed lines mark the significance threshold applied to detect the outlier SNPs (−log(*P* values) = 3).

**Figure 2:**
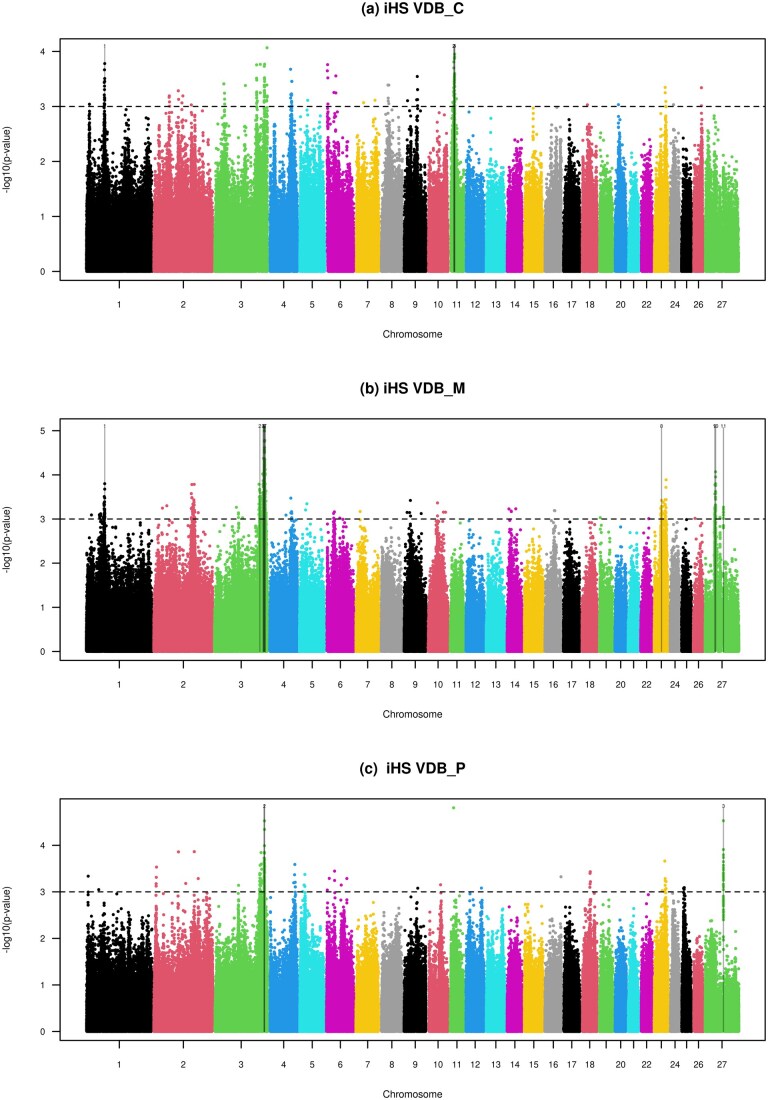
Manhattan plots of the genome-wide iHS test of the three ecotypes of Valle del Belice. The horizontal dashed lines mark the significance threshold applied to detect the outlier SNPs (−log(*P* values) = 3).

Using the iHS test, we detected putative signatures of selection across seven chromosomes in the Sarda ecotypes, identifying 21 candidate regions in SAR_C, 18 in SAR_M, and 11 in SAR_P (Figure 1; Table S2). In contrast, fewer candidate regions were observed in the Valle del Belice ecotypes, with 4 regions in VDB_C, 11 in VDB_M, and 3 in VDB_P, distributed across five chromosomes (Figure 2; Table S2).

Because our primary objective was to identify genomic regions under selection associated with adaptation to mountainous environments, subsequent analyses focused on comparisons involving the mountain ecotypes. To this end, we applied two complementary approaches, iHS and Rsb, to assess whether specific variants were significantly associated with the mountain ecotypes of both breeds. The strongest iHS signals were detected on the X chromosome in SAR_M, where two closely spaced intergenic SNPs showed highly significant values (−log *P* value > 6.5) at approximately 44.6 Mb (Figure 1). In VDB_M, the most pronounced signal was observed on OAR03, where a cluster of 38 SNPs exhibited elevated iHS values (−log *P* value > 4) spanning the 210.52–212.03 Mb region (Figure 2). Within this cluster, we identified a missense variant in the *ELOVL fatty acid elongase 8a* gene (*ELOVL8A*), which catalyzes the elongation of long-chain fatty acids into very long-chain fatty acids. These lipids are essential components of various cellular processes, including lipid metabolism, membrane structure, and cell signaling (Kyselová et al. 2022). The missense variant in *ELOVL8A* gene may thus contribute to adaptive changes in lipid metabolism in response to the colder, energetically demanding mountain environment. Another notable finding on OAR03 is the presence of two additional candidate regions under selection in the mountain ecotypes of both breeds, 166.89–167.09 Mb in SAR_M and 191.26–191.46 Mb in VDB_M. These regions share two key characteristics: 1) they were exclusively detected in the mountain ecotype and were absent from the corresponding plain and hill populations (Table S2), and 2) they overlap with outlier windows previously reported as mountain-ecotype-specific using the medium-density SNP chip (Ben Jemaa et al. 2025). The region specific to SAR_M (OAR03: 166.89–167.09 Mb) overlaps with two long non-coding RNAs (lncRNAs), *ENSOARG00020040868* and *ENSOARG00020030600*, whereas the VDB_M-specific region (OAR03: 191.26–191.46 Mb) encompasses a transcription factor gene, *SOX5*. The lncRNAs are involved in the adaptation of yak to high-altitude environments (Xin et al. 2020) and participate in various physiological processes in bovines, such as immune inflammatory response during infection (Ma 2019; Gupta et al. 2019) and skeletal muscle satellite cell proliferation and differentiation (Sun et al. 2016; Jin et al. 2017; Chen et al. 2019). Muscle satellite cells are a population of stem cells of skeletal muscle that are in a quiescent state in healthy adult muscle and become activated when muscle is damaged. The presence of selective pressure on lncRNAs observed in SAR_M could thus enhance muscle regeneration and/or optimize energy use in muscle. Likewise, *SOX5* is essential for cartilage formation (Smits et al. 2001), skeletal development, and bone healing (Uusitalo et al. 2001; Ikeda et al. 2005), which suggests that variants within this gene in the VDB_M ecotype might lead into robust bones, flexible joints, and faster healing, which are key for survival in rugged terrains.

**Figure 3:**
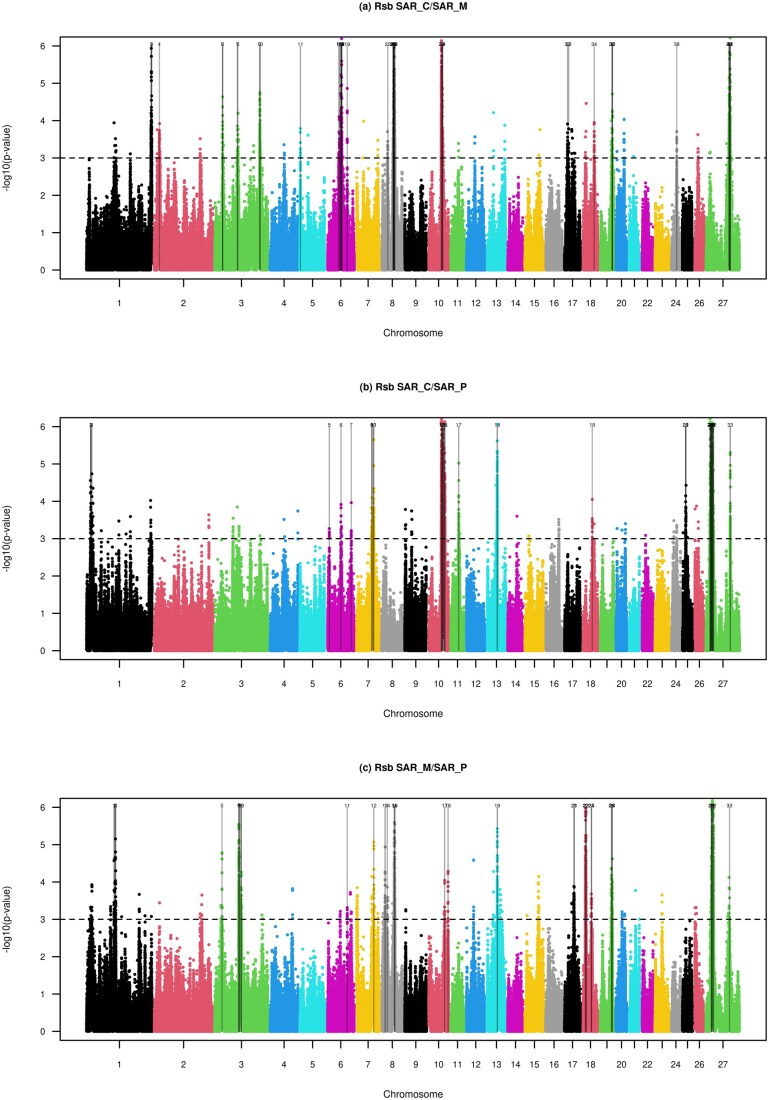
Manhattan plots of the genome-wide Rsb test between the three ecotypes of Sarda. The horizontal dashed lines mark the significance threshold applied to detect the outlier SNPs (−log(*P* values) = 3).

The Rsb test revealed 42, 33, and 33 outlier windows distributed over 16 chromosomes in SAR_C/SAR_M, SAR_C/SAR_P and SAR_M/SAR_P, respectively (Figure 3; Table S3). The SAR_C/SAR_M and SAR_M/SAR_P comparisons detected 26 and 12 candidate regions, respectively, under selection in the SAR_M ecotype (Table S3). Additionally, we found that three genomic regions on OAR03 (33.21–34.25 Mb) and OAR08 (53.43–55.71 Mb; 56.49–57.11 Mb) are under selection in the mountain ecotype in both comparisons (i.e., SAR_C/SAR_M and SAR_M/SAR_P) (Figure 3; Table S3), with the former also shown to be under selection in SAR_M with the medium-density chip (Ben Jemaa et al. 2025). Most of the statistically significant SNPs in these three windows fell within introns of eight protein-coding genes and were classified as modifier impact variants based on the VEP-derived annotations (Table S4). We identified three genes (*RAB10*, *PTPRK*, and *AKAP7*), which exhibit pleiotropic effects on muscle development, as well as on the immune and nervous systems.

By examining all the candidate regions under selection in SAR_M, we found some highly significant missense and synonymous variants (−log *P* value > 5) within *LAMA2* (OAR8: 53.80–55.36 Mb), *SLCO3A1* (OAR18: 12.06–14.76 Mb), and *AKAP13* (OAR18: 15.60–15.98 Mb*)* genes, resulting in low to moderate impact on the protein (data not shown). These three genes are directly involved in several pathways that can be associated with environmental pressures specific to mountainous regions. The *LAMA2* gene plays a critical role in maintaining muscle fiber integrity (Martins et al. 2024) and in supporting vascular and neuromuscular junction function (Previtali and Zambon 2020). *SLCO3A1* encodes an organic anion transporter predominantly expressed in glial cells (Lauterbach 2012), which play a pivotal role in the neuroendocrine regulation of whole-body metabolism (Nampoothiri et al. 2022), whereas *AKAP13* is essential for cardiomyocyte differentiation and heart morphogenesis (Mayers et al. 2010). Another candidate gene identified within a relevant region on chromosome 3 (189.06–190.44 Mb), detected exclusively in the SAR_C/SAR_M comparison, is *LMNTD1*. This gene, associated with respiratory neoplasms, was also highlighted in our previous study (Ben Jemaa et al. 2025). Beyond its role in tumorigenesis, the *LMNTD1* gene may contribute to respiratory adaptation of the SAR_M population. Overall, the selective pressures observed on the aforementioned genes suggest adaptive mechanisms enhancing lung function, cardiovascular efficiency, muscle resilience, and neuroendocrine regulation, which together may promote the development of stronger, fatigue-resistant muscle fibers and improved pulmonary efficiency, facilitating better oxygen uptake and utilization in SAR_M individuals thriving in the rugged, high-altitude environment.

**Figure 4:**
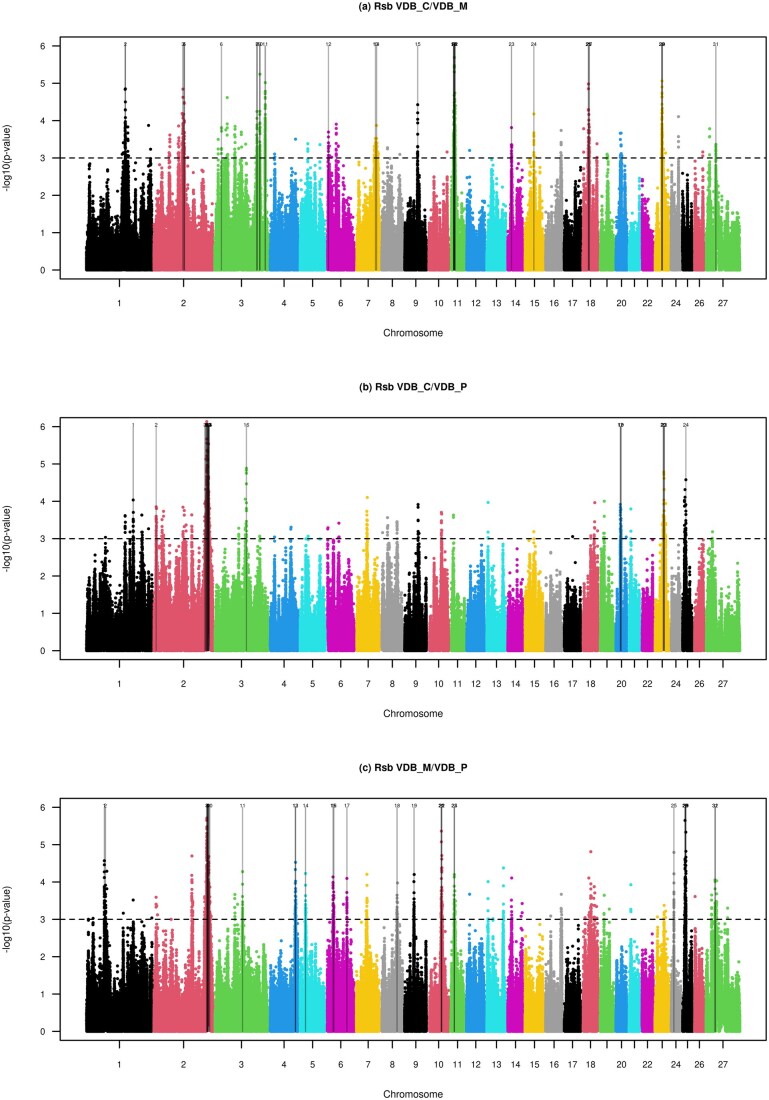
Manhattan plots of the genome-wide Rsb test between the three ecotypes of Valle del Belice. The horizontal dashed lines mark the significance threshold applied to detect the outlier SNPs (−log(*P* values) = 3).

In Valle del Belice, the Rsb test identified 31, 24, and 32 outlier windows in the VDB_C/VDB_M, VDB_C/VDB_P, and VDB_M/VDB_P comparisons, respectively (Figure 4; Table S3). Among these, one of the most relevant signals was detected on OAR02 (221.52–222.65 Mb), showing strong evidence of selection in the VDB_P ecotype. This region was highly significant in both the VDB_C/VDB_P and VDB_M/VDB_P comparisons (Figure 4). The VDB_M ecotype exhibited several selected regions, with eight and five outlier windows detected in the VDB_C/VDB_M and VDB_M/VDB_P comparisons, respectively (Table S3).

As in the case of SAR, we identified several overlaps with the results from the medium-density chip (Ben Jemaa et al. 2025), particularly with the candidate regions on OAR06 (84.63–85.15 Mb) and OAR03 (189.58–190.37 Mb and 211.85–213.17 Mb). The latter region, identified in the VDB_C/VDB_M comparison, showed the strongest signal (Table S3) and harbored several missense and synonymous variants within 10 genes (Table S5). Most of these 10 genes are known to play roles in DNA damage repair (*RHNO1* and *DDX11)*, immune regulation (*FKBP4, CRACR2A, NRIP2*, and *ITFG2*), neurogenesis (*PRMT8)*, and skeletal muscle development (*TEAD4)*, which may have contributed to adaptation to mountainous environment in VDB. Within the candidate region on chromosome 6, we identified a synonymous variant in the t*ransmembrane serine protease 11E* (*TMPRSS11E*) gene, which has been implicated in human lung disease (An and Bain 2023) and shown to facilitate respiratory viral infection by cleaving viral proteins on epithelial surfaces, particularly in the airways (Xiao et al. 2025). Therefore, the synonymous mutation observed in *TMPRSS11E* may contribute to the VDB_M ecotype’s reduced susceptibility to respiratory infections and enhanced epithelial integrity under the cold, pathogen-rich conditions characteristic of high altitudes.

### Complementary and convergent signals between iHS and Rsb tests

The iHS and Rsb statistics are widely regarded as complementary approaches for detecting recent positive selection (e.g., Criscione et al. 2025). Whereas iHS is particularly powerful for identifying selective sweeps at intermediate allele frequencies, Rsb is more sensitive to high-frequency or nearly fixed sweeps (Voight et al. 2006; Tang et al. 2007). Consistent with these methodological differences, we observed limited overlaps between the genomic regions detected by the two methods, suggesting that they capture distinct selective signals and potentially different stages of adaptive processes (Vitti et al. 2013; Bahbahani et al. 2015; Mastrangelo et al. 2020). Nevertheless, a small number of convergent signals were identified in the Sarda breed. Notably, a strong signal on the X chromosome (26.36–28.85 Mb) detected by Rsb in the SAR_M/SAR_P comparison (Table S3) overlaps two SAR_M selected regions identified by iHS (27.37–27.75 Mb and 27.78–28.11 Mb; Table S2). Most significant variants in this interval are intronic within *IL1RAPL1*, a gene with well-studied functions in the synaptic and neuronal functions (Montani et al. 2019). Notably, *IL1RAPL1* expression is downregulated following pharmacological modulation of the glucose-dependent insulinotropic polypeptide receptor, including both agonists and antagonists, and treatments associated with body-weight loss (Gutgesell et al. 2025). Moreover, *IL1RAPL1* shows strong signatures of selection in chickens with large body weight (Wang et al. 2023). Together, these findings suggest that variation in *IL1RAPL1* may contribute to body-size differences and could partly underline the smaller body size observed in the SAR_M ecotype (Cesarani et al. 2022).

The comparison of iHS and Rsb results for the Valle del Belice breed revealed two congruent candidate regions detected in the Rsb VDB_C/VDB_M analysis on OAR23 (31.65–32.09 Mb) and the X chromosome (41.57–41.89 Mb) (Table S3), which overlapped with regions identified by iHS within the VDB_M ecotype (Table S2). All SNPs within the X-chromosome candidate region were intergenic. In contrast, the candidate region on OAR23 (31.65–32.09 Mb) contained 19 significant SNPs, 17 of which were located within introns of *ZNF521*, a transcription factor involved in adipose tissue regulation. *ZNF521* acts as a negative regulator of adipogenesis (Kang et al. 2012; Gustafson et al. 2019) and promotes bone and cartilage formation (Correa et al. 2010). Selection on this gene in the mountain ecotype of Valle del Belice may therefore reflect adaptive modulation of adipose reserves, facilitate energy buffering while maintain locomotion efficiency under extensive grazing conditions. Additionally, selection on *ZNF521* may contribute to increased bone density and skeletal robustness, reducing fracture risk in rugged mountainous environments.

### Impact of marker density on the resolution of selection signals

Although several genomic regions putatively under selection in the mountain ecotypes of Sarda and Valle del Belice sheep were shared between the medium- and high-density SNP datasets, the specific candidate genes identified within these regions differed between the two analyses. This discrepancy most likely reflects differences in marker density. Indeed, the lower number of markers in the medium-density panel reduces the precision with which selection signals can be localized, increasing the likelihood that significant SNPs fall in linkage disequilibrium with causal variants in nearby genes rather than within the actual targets of selection. In contrast, the higher-density dataset provides finer genomic resolution and greater power to detect true associations (Carta et al. 2026), enabling a more accurate identification of the genes directly involved in adaptation to mountain environments.

Despite differences in the specific candidate genes identified, both analyses converged on similar biological pathways likely contributing to adaptation to the mountainous environment in the Sarda and Valle del Belice breeds. In particular, the genes identified with both SNP densities are involved in the regulation of neuroendocrine, nervous, and cardiovascular systems, as well as immune response, respiratory efficiency, and musculoskeletal development, which are key physiological processes supporting adaptation to the demanding conditions of high-altitude environments.

## Conclusion

Using independent samples of Sarda and Valle del Belice sheep and high-density genotypes, we confirmed and refined several candidate regions previously identified with the 50K SNP chip. Because validation of selection signals is often lacking in genomic studies, this work provides a valuable opportunity to evaluate the robustness and reproducibility of previously reported signals using higher-resolution data. Although the specific genes potentially under selection differ between the mountain ecotypes of Sarda and Valle del Belice sheep, they converge on similar biological pathways and functions. Overall, our results underscore the complex, multifactorial nature of sheep adaptation to mountainous environments.

## Supplementary Material

skag055_Supplementary_Data

## Data Availability

The datasets generated and/or analyzed during the current study are not publicly available but are available from the corresponding author upon reasonable request.

## Abbreviations

EHH: extended haplotype homozygosity
his: integrated haplotype score
lncRNAs: long non-coding RNAs
OAR: ovine chromosome
Rsb: standardized log-ratio of integrated EHHS (iES) between pairs of populations
SAR: Sarda sheep
SAR_C: hill ecotype of Sarda sheep
SAR_M: mountain ecotype of Sarda sheep
SAR_P: plain ecotype of Sarda sheep
VDB: Valle del Belice sheep
VDB_C: hill ecotype of Valle del Belice sheep
VDB_M: mountain ecotype of Valle del Belice sheep
VDB_P: plain ecotype of Valle del Belice sheep
VEP: variant effect predictor
